# Endoscopic Local Excision (ELE) with Knife-Assisted Resection (KAR) Techniques Followed by Adjuvant Radiotherapy and/or Chemotherapy for Invasive (T1bsm2,3/T2) Early Rectal Cancer: A Multicenter Retrospective Cohort

**DOI:** 10.3390/jcm13226951

**Published:** 2024-11-18

**Authors:** George Tribonias, Apostolis Papaefthymiou, Petros Zormpas, Stefan Seewald, Maria Zachou, Federico Barbaro, Michel Kahaleh, Gianluca Andrisani, Shaimaa Elkholy, Mohamed El-Sherbiny, Yoriaki Komeda, Raghavendra Yarlagadda, Georgios Tziatzios, Kareem Essam, Hany Haggag, Gregorios Paspatis, Georgios Mavrogenis

**Affiliations:** 1 Department of Gastroenterology, Red Cross Hospital, 11526 Athens, Greece; 2 Digestive Diseases and Surgery Institute, Cleveland Clinic, London SW1X 7HY, UK; 3 Center for Gastroenterology, Hirslanden Clinic Zurich, 8032 Zurich, Switzerland; 4 Department of Gastroenterology, “Sismanogleio” General Hospital, 15126 Athens, Greece; 5 Digestive Endoscopy Unit, Fondazione Policlinico Universitario Agostino Gemelli IRCCS, 00168 Rome, Italy; 6 Department of Gastroenterology, Rutgers Robert Wood Johnson Medical School New Brunswick, New Brunswick, NJ 08901, USA; 7 Digestive Endoscopy Unit, Campus Bio-Medico, University of Rome, 00128 Rome, Italy; 8 Gastroenterology Division, Internal Medicine Department, Faculty of Medicine, Cairo University Kasr Alainy, Cairo 4240310, Egypt; 9 Department of Basic Medical Sciences, College of Medicine, AlMaarefa University, Riyadh 13713, Saudi Arabia; 10 Department of Gastroenterology and Hepatology, Faculty of Medicine, Kindai University, Osaka-Sayama 589-0014, Japan; 11 Asian Institute of Gastroenterology—Gastroenterology, Hyderabad 500082, India; 12 Department of Gastroenterology, “Konstantopoulio-Patision” General Hospital, 14233 Athens, Greece; 13 Gastroenterology Division, Department of Internal Medicine, Faculty of Medicine, Cairo University, Cairo 4240310, Egypt; 14 Department of Gastroenterology, Venizeleion General Hospital, 71409 Heraklion, Greece; 15 Unit of Hybrid Interventional Endoscopy, Department of Gastroenterology, Mediterraneo Hospital, 16675 Athens, Greece

**Keywords:** rectal cancer, infiltrative polyp, ESD, EID, EFTR, knife-assisted dissection, colonoscopy

## Abstract

**Background:** Resected rectal polyps with deep invasion into the submucosa (pT1b-sm2,3) or the muscle layer (pT2) are currently confronted with surgery due to non-curative resection. **Aims:** We evaluated the efficacy, safety, and locoregional control of adjuvant radiotherapy (RT) and/or chemotherapy (CT) following endoscopic KAR (knife-assisted resection) in patients with invasive early rectal cancers who are unwilling or unsuitable for additional surgical resection. **Methods:** Fifty-one patients with early rectal cancers, pT1b or pT2, underwent post-resection adjuvant RT and/or CT in 15 centers worldwide. “En bloc” macroscopic resection, R0 resection, recurrence rate, and adverse events following resection and adjuvant therapy were recorded in a multicenter retrospective cohort study. **Results:** Diagnostic staging (38/51, 75%) was the main reason for ELE. Macroscopic “en bloc” resection was demonstrated in 50/51 (98%), with an average follow-up of 20.6 months. Endoscopic recurrence occurred in 7/51 (13.7%) of patients, with mean time for diagnosis of recurrence at 8.9 months. Adjuvant therapy consisted of RT in 49.0% (25/51), CT in 11.8% (6/51), and combined CRT in 39.2% (20/51) of the cases. Perforation, severe post-procedural bleeding, and incontinence were the most frequent complications. The absence of superficial ulceration was associated with macroscopic complete resection, while the lesions with lower budding stage, clear lateral margins, lesion size < 40 mm, and needle-type knife used were associated with less endoscopic recurrencies. **Conclusions:** Our data investigated adjuvant RT and/or CT after endoscopic KAR of infiltrative rectal cancers (pT1bsm2,3-pT2) as being safe and effective for locoregional control and providing a non-surgical treatment option for patients with a non-curative resection.

## 1. Introduction

Early rectal cancer has lately been a subject of debate in terms of management, given the fact that the mainstay therapy is neo-adjuvant chemoradiation (CRT) followed by Total Mesorectal Excision (TME) as the gold standard to achieve local control and disease cure [1,2]. However, TME poses significant perioperative mortality, short- and long-term morbidity risks, and also negatively affects patients’ quality of life (QoL) [3]. Numerous studies have demonstrated that local excision (LE) by surgical techniques (TEM/Transanal Endoscopic Microsurgery, TAMIS/Transanal Minimally Invasive Surgery) after adjuvant radiotherapy (RT) and/or chemotherapy (CT) provides safe short-term alternatives regarding locoregional control and QoL [4,5,6,7]. Whilst improving certain parameters LE based on surgical techniques, given that it’s almost always a full thickness bowel wall resection, it can complicate subsequent salvage surgery, which may be indicated in cases of inadequate oncological control [8]. In addition, certain tumor characteristics such as distance from the dentate line and tumor size may hinder or even restrict LE by surgical techniques.

Endoscopic knife-assisted resection (EKAR) techniques, either in the submucosal plane (Endoscopic submucosal dissection (ESD)), intermuscular plane (Endoscopic intermuscular dissection (EID)), or even full-thickness resection (endoscopic full thickness resection (EFTR)) may prove to be adequate and applicable to a wider range of patients in need of LE. The European Society of Gastrointestinal Endoscopy’s (ESGE) guidelines on ESD in rectal lesions states that when deep infiltration (>1000 μm) is confirmed, this should be considered as a high risk (non-curative) resection, because the risk of lymph node metastasis (LNMs) exceeds 3% and, therefore, additional treatment should be sought [9]. It is of note that if complete resection is documented and sm2 invasion is the only high-risk criterion, further therapy might carry greater risk than surveillance alone [9,10]. In early T1b and T2 rectal cancer, where the risk of lymphatic spread is low, adopting surgery by either low anterior resection (LAR) or abdominoperineal resection (APR) may lead to overtreatment, particularly as TME carries morbidity, risks of complication, and involves the formation of a temporary or permanent colostomy. It is noteworthy that the rates of local control following LE based on topical, minimally invasive resections have been shown to be favorable in Tis, T1 sm1–2 tumors [11].

Based on the emergence of endoscopic resection techniques for LE in benign rectal neoplasms and early malignant lesions during the last decades (ESD, EID, and EFTR) [12,13,14] and the growing interest in organ-sparing treatment options, we conducted this multicenter study in order to evaluate the efficacy of endoscopic local excision (ELE) following adjuvant RT and/or CT for patients diagnosed with lesions with deep submucosal invasion into the mid-lower third of the submucosa (pT1b-sm2,3) or the muscle layer (pT2), who were unwilling or unfit to undergo surgery. Although advanced EKAR techniques have been tested for the excision of residual carcinomas after CRT [15], data on ELE followed by CRT are lacking in the literature. The principal aim of this study intended to answer this question by evaluating recurrence rates for ELE with concurrent RT and/or CT in invasive early rectal cancers (pT1b, T2), with respect to established and potential risk factors, and guide patient selection for endoscopic management.

## 2. Materials and Methods

Fifteen centers around the world dedicated to advanced endoscopic resection techniques participated in this retrospective multicenter study by allotting their relevant records from 2019 to 2023. The study was based on the Strengthening the Reporting of Observational studies in Epidemiology (STROBE) guidelines [16]. A structured protocol, which corresponded to the ethical guidelines of the last revision of the Declaration of Helsinki and complied with Good Clinical Practice Guidelines [17,18], was approved by the Scientific Committee of the main coordinating center and was the reference for all involved centers. Patient anonymity was ensured and the data received were de-identified.

**Figure 1:**
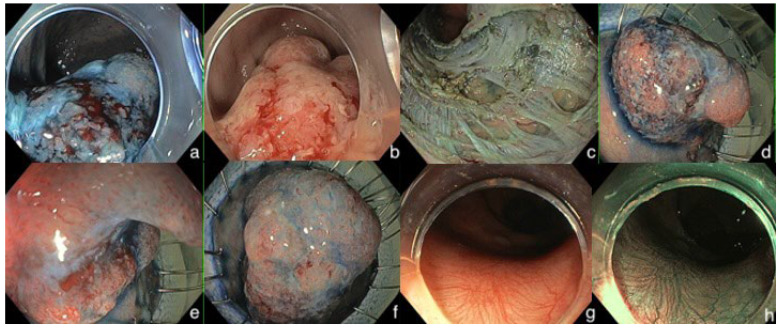
Endoscopic images of a pT1bsm3 invasive early rectal cancer removed by endoscopic KAR (knife-assisted resection) technique deeply in the muscularis mucosa with partial EFTR (endoscopic full-thickness resection). (**a**–**f**) refer to the resection part. (**g**,**h**) refer to the endoscopic surveillance, 12 months after dissection.

Eligible patients were aged 18 years or older; able to receive RT +/− CT; and had an early rectal cancer, pathologically staged pT1 or pT2, and clinical N0 M0. The patients underwent an EKAR technique (ESD, EID, or EFTR) for resection of an advanced rectal polyp due to diagnostic reasons, unfitness for surgery, or patient’s preference (Figure 1). After removing the polyp and classification of the resection as a “non-curative” one, according to the recent ESGE guidelines [9], a Multidisciplinary Team (MDT) meeting proposed adjuvant RT or CT or combined CRT based on patients’ eligibility and willingness for surgery. Exclusion criteria included patients who had received prior rectal cancer treatment; non-adenocarcinoma histology; those with metastatic disease, synchronous or previous malignancy; early rectal cancers other than N0 and M0; patients who received brachytherapy; indefinite or missing data; and absent follow up. In all recruited patients, the dissection had to be executed with an ELE technique based on a knife-assisted resection (KAR) method, excluding other snare-based techniques (EMR/Endoscopic Mucosal resection; Hot and Cold snare polypectomy).

Cases fulfilling the eligibility criteria were recruited. The appropriacy of inclusion was evaluated by GT, PZ, and AP. The following variables were retrieved: (1) demographics’ clinical parameters (age at diagnosis, sex, and ASA score [19]); (2) endoscopic features of the lesion (location, size, superficial morphology, and electronic chromoendoscopy findings in magnification with appropriate classifications); (3) duration and details of the resection; (4) complications (bleeding, perforation, pain, and incontinence); (5) histologic findings (size of specimen; cancer subtype; budding score; and submucosal, lymphovascular, perineural, or vascular invasion); (6) preoperative staging depictions; (7) recurrence and duration of follow up; (8) adjuvant chemoradiotherapy after resection/complications; (9) postoperative follow-up depictions.

Recently, emerging data from surgical publications [20,21,22,23] underlined the option of adjuvant pelvic RT for patients with early invasive rectal cancers who had been initially managed with LE. Patients with an early rectal cancer (T1b-T2) and “non-curable” resection, due to margin positivity/very close margin (<1 mm) at time of local excision or having 2 or more risk factors for locoregional recurrence—depth of invasion > 1 mm, high grade adenocarcinoma (G3–G4), lymphovascular invasion, perineural invasion, tumor budding > 1, or mucinous subtype—referred to an oncological MDT in order to explore a radical TME surgery with LAR or APR or to investigate any alternative option [9]. An MDT meeting, after reviewing the final histopathological results of LE, concluded whether the patient was suitable for adjuvant CRT or radical surgery according to comorbidity, patient’s preference, endoscopist’s experience, and risk factors of tumor.

Patients were clinically monitored by their oncologist weekly during their pelvic radiation therapy and then at 4 weeks post-treatment. Thereafter, they were followed up clinically at 3-month intervals with 6-monthly surveillance colonoscopy and MRI imaging during the first year. Afterwards, they underwent yearly colonoscopy and imaging with biannual clinical follow-up. The primary endpoint of interest was the technical success of EKAR techniques, loco-regional control, and recurrence rate. Secondary outcomes included assessment of potential risk factors associated with recurrence, determination of the time of recurrence, treatment-related toxicity, and assessment of resection-related adverse events. Kaplan–Meier methods were not employed for estimation of survival endpoints because none of the recruited patients died during the follow-up period.

An Excel file (Microsoft Excel for Mac 2019, Microsoft Corporation, Redmond, WA, USA) with predetermined available variable values was created and shared with the involved centers. All data were stored on a secure server.

### Statistical Analysis

Data analysis was performed using the Statistical Package for Social Science Software for Windows (IBM SPSS Statistics, Version 28.0. Armonk, NY, USA: IBM Corp). Continuous variables are presented as mean (±standard deviation) or median (IQR), and categorical variables are shown as percentages. Recurrence after resection over time was calculated according to the Kaplan–Meier method. The log-rank test was performed for analysis. Univariable models were used to investigate individual associations between independent variables and the ability for “en bloc” resection, recurrence, and complications, while in the multivariable regression, all variables were inserted to assess their relationship with these parameters. *p* ≤ 0.05 (two tailed) was considered statistically significant. Tables were created using R programming language and gtsummary package.

## 3. Results

After applying the inclusion and exclusion criteria, 51 cases (Switzerland: fourteen, Greece: twelve, Italy: ten, USA: five, Egypt: five, India: two, Japan: two, and Saudi Arabia: one) from 15 advanced endoscopy centers worldwide were considered as participants. All the cases had been executed by endoscopists highly experienced in ESD and KAR endoscopic procedures. Table 1 summarizes the main characteristics of our sample. The cases were accomplished between 21 January 2019 and 31 May 2023, and the data were collected retrospectively. The male to female ratio was 30/21 (59%/41%) and the mean age was 65.0 ± 11.1 years. The higher percentage of the patients was classified, according to the American Society of Anesthesiologist’s physical status classification system, as ASA 1 (16, 31.4%), ASA 2 (23, 45.1%), and ASA 3 (11, 21.6%), with only one patient being included in the ASA 4 category (1, 2%). The vast majority of the patients underwent endoscopic resection for diagnostic–staging reasons 38/51 (75%), with 18% performed according to patient’s preference and 7.8% being unsuitable for surgery due to comorbidities.

Table 2 describes and analyzes the lesions’ characteristics. The most frequent site of early rectal cancer in our cohort was the lower rectum, with 58.8% (30/51), followed by the mid and the upper rectum (13/51 and 8/51, respectively). A total of 35 lesions were resected from the anterior (68.6%) and 16 from the posterior rectal wall (31.4%). The mean distance from the dentate line was 3.9 ± 3.6 cm. The mean size of the tumors, as assessed by the endoscopists, was 45 ± 26 mm, with 88.2% of them > 20 mm. A size for resected lesions > 40 mm was significantly related to the presence of recurrence (*p* = 0.011), since all the cases with a recurrent adenoma or carcinoma referred to polyps > 40 mm. Considering the mucosal classifications under image-enhanced endoscopy (IEE-NBI/Narrow Band Imaging, Olympus, Tokyo, Japan) and morphological features according to Paris Classification [24] and LST (Lateral Spreading Tumor) Classification [25], the majority of the lesions were classified as JNET (Japanese NBI Expert Team) [26] Classification: 2B or 3, 45/51 (80.4%), sessile (Paris Classification: 0-Is) or with major sessile component (Paris Classification: 0-IIa + Is), 34/51 (66.7%), and LST/Granular-type (32/51, 62.8%) of the lesions, respectively. The main characteristics for submucosal invasion were the presence of depression (16/51, 31.4%) or ulceration (8/51, 15.7%). Interestingly, in 76.5% (39/51) of cases, the endoscopists had a histology result before the execution of the procedure. Preoperative histological diagnosis of cancer (Intramucosal or Infiltrating submucosal) was demonstrated in 41.0% (16/39, *p* = 0.017), with the rest of the biopsied lesions (23/39, 59.0%) diagnosed either as low- or high-grade dysplasia. MRI with rectal protocol and rectal EUS (ERUS) for staging had been performed in 45.1% and 37.3%, respectively. Moreover, MRI, ERUS, and simple biopsy sensitivity in staging compared to histology on resection were 81.8% (48.2–97.7%), 87.5% (61.7–98.5%), and 36.1% (20.8–53.8%), respectively.

Table 3 presents the resection characteristics and perioperative variables. Specifically, the mean duration for the endoscopic LE was 186 ± 136 min and the mean size of the resected specimens, measured by the pathologists, was 61 ± 28 mm. The majority of the patients underwent resection under general anesthesia (80.4%), with the rest of them under deep conscious sedation supervised by an anesthesiologist. Although ESD was the most frequently implemented technique (76.5%), the resection plane was executed deeper in 11/51 cases with EID and with partial and complete EFTR. With a view to the resection’s details, the most common endoscopic knives used were needle-type knives (Dual knife, Flush knife, or Hybrid knife) in 76.5% of cases. Both needle-type knives and the Hook knife (13.7%) were more effective (*p* < 0.001) regarding the development of recurrence during follow-up compared to insulated-type knives (IT-nano, IT2). Closure of the defect was executed only in 8/51 (15.7%) cases with either Through-The-Scope/TTS Clips or a combination of an elastic loop (Endoloop) and TTS clips. Additionally, macroscopic complete (“en bloc’’) resection was reported, by the endoscopists, in the majority of the cases (50/51, 98%). The absence of an ulceration positively affected the macroscopic complete resection (*p* = 0.019). However, the R0 resection rate, according to the pathologists, was demonstrated in 31/51 (60.8%) of the cases because of positive lateral margins (LM+) or positive vertical margins (VM+) in three (5.9%) and nineteen (37.3%) cases, respectively. Even though R0 resection in pT2 cancers was achieved in half of the patients (4/10), endoscopic recurrence surfaced only in one pT2 case. Regarding pathology reports, infiltrative classical adenocarcinoma was revealed in 92.2% of cases, with pT1bSM2 being the most common tumor stage (39.2%) and 2175.6 ± 932.3 μm being the mean infiltration depth from muscularis mucosa. Most of the lesions were characterized as early rectal cancers with good differentiation (G1 + G2 tumors, 78.4%), low budding score (Bd1) in 29/51 (56.9%) cases, and absence of lymphovascular infiltration and perineural invasion in 35 (68.6%) and 48 (94.1%) cases, respectively. The lesions with lower budding stages and clear lateral margins in histology were associated with less endoscopic recurrences (*p* = 0.050 and *p* = 0.011, respectively). Resections were completed without a complication in 41/51 cases (*p* < 0.001). The recorded complications—perforation 5.9% (3/51), incontinence 3.9% (2/51), severe post-procedural bleeding 3.9% (2/51), and pain 3.9% (2/51)—presented with a higher trend during the resection of lesions in the mid rectum (*p* = 0.073). None of the complications imposed patients to seek further surgical intervention in order to deal with them. The variables providing significance in chi-square test were included in binary logistic regression analysis models to investigate potential associations with “en bloc” resection or recurrence; however, they failed to provide statistical significance, probably due to the limited sample size.

After resection, the patients were followed-up with structured endoscopic surveillance for a mean time period of 20.6 ± 15.8 months (Table 4). Overall, endoscopic recurrence was detected in 7/51 (13.7%) patients, four (7.8%) of them with carcinoma recurrence and three (5.9%) with adenoma recurrence. Moreover, the mean time of recurrence detection was 8.9 ± 8.8 months, with endoscopic resection of the recurrent lesion being the most frequent treatment of choice. During follow-up, 5/7 (9.8%) of recurrences were detected in rectal MRI, whereas one patient was detected with lymph node metastasis and two with concomitant distant metastasis in abdomen CT scan. Adjuvant therapy following endoscopic resections consisted of RT alone in 49.0% (25/51), CT alone in 11.8% (6/51), and a combination of CRT in 39.2% (20/51) of the cases. Additional CT had been offered to almost all patients (6/7), which demonstrated recurrence during follow-up (*p* < 0.001), reflecting the oncologists’ prediction for recurrence. Overall, the vast majority of the patients (88.2%) received additional radiotherapy, with three reported cases with blood per rectum and pain due to radiation proctitis and one with post-radiation stenosis. The most frequent chemotherapy schema was capecitabine (15/51, 29.2%) and the mean radiation schema was 48 ± 1.5 Gy. In the RT or CRT group, all patients received 46–52 Gy (EQD2) to the primary and 43–48 Gy (EQD2) to the pelvic nodal regions, with 32 of the 45 patients (71.1%) treated with 3D conformal radiation therapy (3DCRT). The remaining patients (5/51) were not suitable for radiotherapy due to comorbidities and/or performance status or declined concurrent radiotherapy; as a result, they were treated with chemotherapy alone. The MDT meeting proposal (43%) and patient willingness (57%) were the reasons for no additional surgical treatment. Nevertheless, one case was additionally treated with TAMIS for topical excision of a carcinoma recurrence.

Table 5 explores the statistical relations of assessed variables based on the criterion of endoscopic recurrence. Variables that demonstrate statistical significance predictive of endoscopic recurrence are the type of knife used (*p* = 0.001), Budding score (*p* = 0.023), lateral margins on histology (*p* = 0.046), follow-up rectum MRI (*p* < 0.0011) and follow-up abdomen CT (*p* = 0.025), and lesion size (*p* = 0.011). However, adjustment for confounders by performing binary regression analysis did not yield statistically significant results. We present these results in Table 6, demonstrating potential associations between endoscopic recurrence and independent variables.

**Figure 2:**
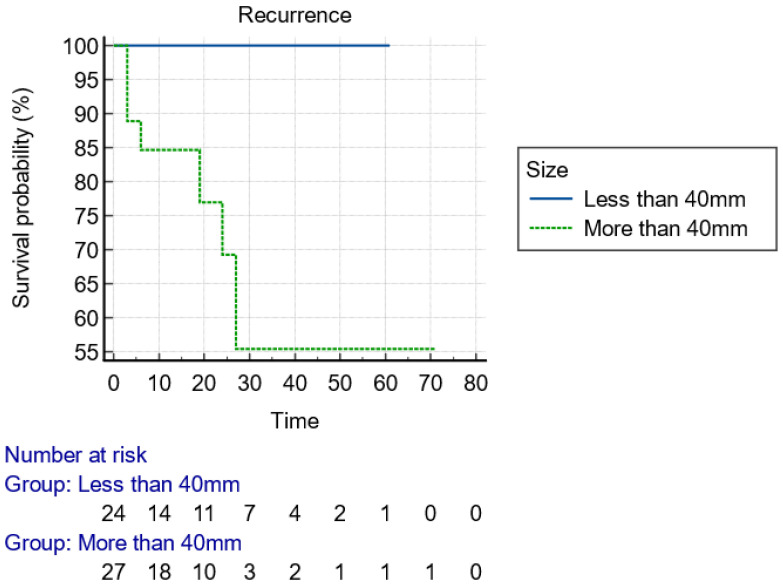
Kaplan–Meier curve presenting the time of recurrence of the lesion with regard to the size, using 40 mm as a cut-off value.

**Figure 3:**
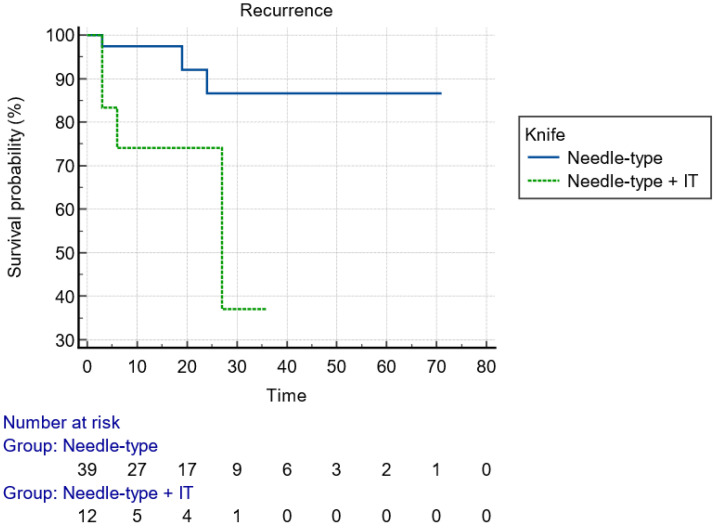
Kaplan–Meier curve presenting the time of recurrence of the lesion with regard to the type of used knife.

**Figure 4:**
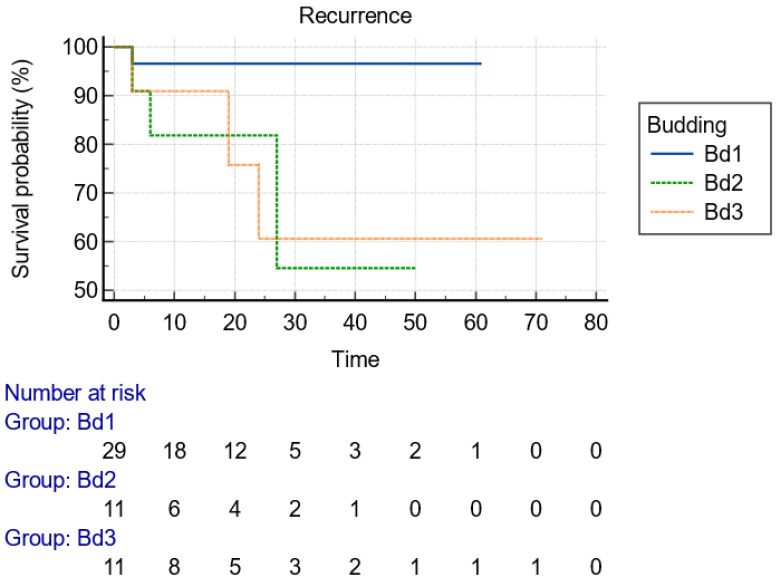
Kaplan–Meier curve presenting the time of recurrence of the lesion with regard to the stage of budding, as presented in histology after resection.

With regard to the size of the lesion (cut-off diameter of 40 mm), the Kaplan–Meier curve indicated that recurrence could happen at any time within the first 2 years (Figure 2). Considering the type of knife, the recurrences were diagnosed early postoperatively when IT knives were used, compared to later recurrences with single use of needle knives, indicative of larger malignant tumor remnant islands into the dissection plane (Figure 3). Finally, advanced budding (Bd3) was associated with recurrences mainly after 18–24 months, whereas in lower stages, recurrences were found within the first 6 months (Figure 4). However, the small and underpowered sample size does not allow safe conclusions on these observations.

## 4. Discussion

TME +/− neoadjuvant therapy remains the cornerstone of therapy for early rectal cancer, associated with a decreased incidence of local recurrence and subsequent improvements in patient survival [27]. Although the management of rectal cancer has improved over the past few decades, no conclusive criteria have yet been established about whether the patients should undergo LE or radical resection [27,28]. The selection of the most suitable type of resection based on accurate staging during the preoperative period remains a challenge. Our findings suggest that LE, accomplished with EKAR techniques and followed by adjuvant pelvic RT and/or CT, is feasible, safe, and yields good locoregional control in T1/T2 N0 M0 rectal adenocarcinoma, thereby avoiding radical surgery in the form of AR or APR. The study encompasses worldwide data and is the first one that exclusively investigated EKAR techniques without surgical alternatives in LE and without being limited to a specific subpopulation or region. The overall local recurrence rate was measured as 13.7%, with carcinoma recurrence detected in four patients and adenoma recurrence in three patients at a median follow-up of 20.6 months (Table 4). Additionally, the mean time of recurrence detection was 8.9 months diagnosed by endoscopic surveillance, while 5/7 recurrences were demonstrated in rectal MRI simultaneously. During follow-up, one patient was detected with lymph node metastasis and two with concomitant distant metastasis in an abdominal CT scan.

After conducting an endoscopic biopsy along with endoscopic diagnosis based on electronic chromoendoscopy, we implemented ELE in patients with clinical and radiological T1 rectal cancer in order to accurately determine the underlying pathology and acquire a verified T-stage. In agreement with previous publications, the biopsy before resection underestimated the malignant potential of lesions, with 63.9% (46.22–79.18%) of the lesions being diagnosed either as low- or high-grade dysplasia preoperatively [29]. On the contrary, the optical diagnosis (JNET-Classification: 2B or 3) more accurately predicted the presence of adenocarcinoma in 80.4% (60.46–87.12%) of the cases. The limitations of current staging methods constitute a real challenge in rectal adenocarcinoma management. Overtreatment with rectal MRI is another confounding factor when examining both the sensitivity and specificity of rectal cancer staging using rectal MRI as a diagnostic tool [30,31]. This is particularly notable in patients with early-stage disease. In a Dutch population-based study, even though a large number of tumors were deemed to be early stage (cT1 to T2N0) [31], and therefore potentially suitable for local excision (LE), pathology reports ultimately revealed half of them to be falsely overstaged, with an incorrect T stage. In our study, the main reason for initial endoscopic resection of rectal polyps was the diagnostic staging of the disease 38/51 (75%), with the majority of dissections accomplished by ESD. Initial staging is, thus, critical to patients for the optimal selection of the most appropriate and least life-altering modality that will cure their malignancy. Until novel strategies emerge that will more accurately stage patients with rectal adenocarcinoma using radiomics [32], definite histological diagnosis and T-staging with EKAR techniques still remains the safest way to overcome disparity between clinical and pathologic staging. A critical and unmet need in the management of rectal adenocarcinoma via an oncologic MDT is the improvement in pretreatment staging, which is essential for selecting the optimal adjuvant therapeutic approach either preoperatively or after LE.

Implementation of screening programs for colorectal cancer has resulted in a notable rise in the identification of colorectal cancer cases during early stages [33,34]. The primary objective in the treatment of patients diagnosed with rectal cancer is to maximize their chances of recovery while also preserving their overall well-being and QoL. The optimal surgical management strategy can range from no surgery at all, to LE, to TME approaches including an APR or an ultra-low LAR with coloanal anastomosis. Avoiding unnecessary surgery and preserving the rectum are important, as the associated risks and morbidity are considerable. On the other hand, the implementation of contemporary minimally invasive EKAR techniques, such as ESD, which is emphasized in organ-sparing theory, entails the knowledge of the advantages and limits of each technique. Indeed, the desirable macroscopic complete resection with EKAR techniques could be hindered from several tumor’s characteristics. In our cohort, the absence of an ulceration on the surface of the polyp positively affected the macroscopic complete resection (*p* = 0.019) of the lesions. ESGE recommends considering ESD for “en bloc” resection of colorectal lesions, particularly in the rectum, when there is suspicion of limited submucosal invasion (characterized by a depressed area with irregular surface pattern or a large protruding or bulky component, especially for lesions larger than 20 mm) or when snare-based techniques are insufficient for complete removal [9]. However, the primary concern unaddressed by conventional ESD revolves around patients with colorectal carcinoma (T1 sm2 or sm3) characterized by deep submucosal invasion. Within this particular group, an estimated 10 to 50% of patients manifest lymph node metastasis, which is closely associated with the presence of histological high-risk features [35]. These features include lymphovascular invasion, aggressive tumor budding, poorly differentiated (grade 3) cancer, or invasion of the vertical margin > 1 mm [9].

Oostendorp et al. [36], in a large meta-analysis, showed a local recurrence rate of 6.7% for low-risk pT1 tumors and 13.6 for high-risk pT1 tumors treated with LE alone. In pT1 patients treated with adjuvant CRT, local recurrence rates were lower. Additionally, in pT2 tumors, local recurrence rates were significantly higher at 28.9% for LE alone vs. 14.7% following adjuvant RT with concurrent chemotherapy [36]. A variety of mostly surgical LE techniques were employed in the previous study, while endoscopic resection modalities were not investigated and described adequately. In our retrospective analysis, the first in the literature exclusively executed with EKAR techniques, the overall endoscopic recurrence was detected in 13.7% of patients (pT1, T2). Even though R0 resection in pT2 cancers was achieved in less than half of the patients (40%), endoscopic recurrence was revealed in only one pT2 case (1/10). Moreover, the mean time of recurrence detection was 8.9 months, with endoscopic resection of the recurrent lesion being the most frequent treatment of choice. Additionally, in our analysis, the rate of distant metastases disease was demonstrated at 3.9%. The two patients in the current study who had distant recurrences both revealed pT1SM3 disease, and the histopathology of their primary tumor demonstrated high budding score. It is reassuring to note that they remained disease-free in the treated pelvis and that their distant relapse probably would not have been prevented in case they had received radical surgery. Although the specific reasons for the distant recurrence in these patients, with the absence of any locoregional recurrence, are not explicitly outlined, it is believed that the treatment approach is not likely to affect the likelihood of distant metastasis. However, aspects such as tumor biology and probably the presence of high-risk features may influence the risk of distant metastasis [37,38]. Due to the retrospective multicenter fashion of the study, the homogenization of the sample was difficult; so, brachytherapy as an adjunctive tool after resection was not included in the analysis. However, it could be an interesting factor to investigate in a future prospective study.

To date, no specific variables have been assessed to provide a reliable predictor of recurrence after EKAR techniques for early invasive rectal cancers. In our analysis, the size of the lesion (*p* = 0.011), the type of ESD knife used (*p* < 0.001) for the resection, the presence of high budding score (*p* = 0.023), and the positive lateral margins (*p* = 0.046) in the resected specimen were significantly associated with the development of recurrence during follow-up. More comparisons based on endoscopic recurrence can be seen in Table 5. Nevertheless, it is known that vascular invasion is a significantly poor prognostic factor for DFS (Disease-Free Survival, *p* = 0.033), and the presence of three or more high-risk features was associated with poor DFS (*p* = 0.002) [21]. Moreover, Nascimbeni et al. [39] reported that the depth of invasion and LVI were associated with a significant risk of lymph node metastasis. We demonstrated that EKAR techniques deal with greater difficulty giant rectal polyps > 40 mm, which are more likely to get inadequately resected with positive lateral margins. The use of an IT-type knife during the resection does not sufficiently enhance the thoroughness of dissection into deeper layers, in comparison with needle-type knives and Hook knife, leading to recurrence emergence (Figure 3). The designing of IT-knives provides more safety, without adequate competency for the dissection of the muscle layers in need of deeper resection. Additionally, a higher Bd score (2,3) is a well-known high-risk feature [35] that predicts lymph node metastases and recurrences in rectal cancer, as also corroborated in our analysis (*p* = 0.050). Oncological MDT is more likely to deliver RT or additional surgical resection in patients with Bd 2,3. Regarding the types and rates of complications, macroscopic complete resection completed significantly without an adverse event during ΕLE-KAR techniques in 80.4% of cases (*p* < 0.001). Complications recorded were perforation in the higher rate (5.9%), incontinence, severe post-procedural bleeding and pain, with a trend but non-significant predilection to the mid rectum (*p* = 0.073). Hospitalization with major surgical intervention was not necessary for any patient with an adverse event.

For patients with T2, N0 rectal cancer, chemoradiotherapy with oxaliplatin and capecitabine (CAPEOX) followed by local R0 excision may be a safe alternative to transabdominal resection [40]. A meta-analysis suggests that the approach of neoadjuvant CRT followed by LE may be a safe and effective alternative for patients with any T and any N stage of rectal cancer who refuse or are unfit for transabdominal resection [22]. The panel of experts advises considering LE as a palliative strategy for elderly patients who are deemed unsuitable for extensive surgical procedures, with neoadjuvant therapy followed by organ-sparing transanal LE for elderly patients with small cT2/T3 N0 M0 early rectal cancers. However, this statement is characterized with evidence of moderate quality [41]. Our study population consists of patients within the whole age range, not only elderly individuals, in which the management differs from the approach taken with the general population in terms of prioritizing outcomes and setting goals for the overall treatment strategy according to age and life-expectancy. Another strength based on our cohort sample size is the fact that all the resections were accomplished homogenously with EKAR techniques, instead of mixed surgical methods (TEMS, TAMIS) or snare-based endoscopic techniques, as was reported in older publications. Surgical minimally invasive resections lead almost always to a full thickness bowel wall resection and may complicate the surgical plane for a subsequent salvage oncological surgery, which may be indicated in cases of inadequate oncological control [28]. Additionally, TEMS and TAMIS are restricted for tumors < 4 cm, occupying < 40% of the rectal circumference and <10 cm from the dentate line [28]. On the contrary, ELE techniques not only preserve the surgical plane by dissecting inside the bowel wall but also deal efficaciously with polypoid lesions that extend up to the dentate line or overthrust it. Surgical LE techniques are also restrained by the implementation of the surgical port onto the dentate line, with a negative influence on the capability of the technique for resection close to it.

Newer studies like the OPERA trial, incorporating contact x-ray brachytherapy in the quiver of neoadjuvant therapies, have shown increased organ preservation rates compared to standard chemoradiation treatment [42]. This watch and wait approach may minimize interventional risks but needs to be evaluated in prospective studies designed to compare the different treatment modalities in combination with brachytherapy. The STAR-TREC trial, which is now in phase III, will provide substantial information in this field, adding to the long-term efficacy of (chemo)radiation and the ‘watch and wait’ strategy directly compared to radical surgery [43].

In the current management of early rectal cancer, surgery is the primary approach. However, European Guidelines recommend considering alternative strategies, such as Transanal Endoscopic Microsurgery (TEM), Chemoradiotherapy (CRT), or a ‘watch and wait’ approach, particularly for patients with early-stage disease (T1–T2, T3a/b) who are frail or at high surgical risk. Comparing CRT toxicity with the morbidity associated with radical surgery is complex due to the distinct nature of complications that each treatment option presents. Taking into account patients’ preference is crucial in this setting, where different kinds of side effects cannot be compared in a straightforward, measurable way. Available data exist on the surgical morbidity differences between LE and radical surgery. Lyyn et al. showed that patients receiving CRT + LE had a lower rate of complications requiring reoperation compared to just the TME group from the Dutch TME trial [44]. CRT + LE patients manifested therapy-related toxicity in 43%, while 51% of TME patients required a permanent stoma. Teste et al. published a post hoc analysis from a randomized trial comparing early and late surgical morbidity in patients receiving CRT + LE, CRT + LE with eventual completion TME, and CRT + TME, showing a significantly lower severity of overall morbidity in the LE group [45]. Additionally, Pacevicius et al. found that, compared to TME, CRT + LE offers a viable alternative for early rectal cancer, reporting reduced complication rates, lower incidence of minor Low Anterior Resection Syndrome (LARS), and shorter hospital stays, without compromising survival outcomes [46].

This study has a number of weaknesses that need to be put into perspective. First, the retrospective single-arm design poses limitations to the generalizability of these findings. Prospective and comparative studies in this setting are challenging, given that guidelines for early invasive rectal cancer in most Western countries recommend conventional surgical techniques, particularly APR and LAR, usually combined with neoadjuvant chemoradiotherapy. Furthermore, patient selection bias needs to be taken into account. Patients receiving LE treatment may have had various comorbidities, may have been less ideal surgical candidates, or may have rejected stoma formation of alternative treatments. Since all involved centers adhered to these recommendations, sample size volume restriction might have impacted the associations observed between certain variables and recurrence, particularly in multivariate regression analyses. Another limitation of this study is the lack of standard departmental policy regarding the surveillance and follow-up methods during the study time frame. Therefore, variations could exist due to clinician or patient preference alongside investigational-cost-related reasons. As a consequence, the follow-up was not uniform regarding the duration and frequency between the participating centers, which may render the evaluation of the long-term therapeutic outcome of LE with or without CT followed by RT less reliable.

Despite these limitations, we believe that our results contribute significantly to the management of early rectal cancers, especially in those whose tumors were incidentally identified during LE for presumed benign or non-neoplastic lesions. Further studies should focus on prospectively evaluating histological and molecular biomarkers predictive of recurrence and response to radiotherapy to further improve the therapeutic strategy in early rectal cancer.

## 5. Conclusions

In the current analysis, we demonstrated the efficacy, safety, and outcomes of a 2-year follow-up, for a treatment approach combining EKAR techniques with adjuvant RT or/+ CT for patients with T1,2 N0 M0 rectal cancer who had non-curative resections, were deemed unsuitable for surgery, or preferred non-surgical interventions. To conclude, ELE with EKAR techniques, even being “non-curative” based on the current recommendations, seems to have a role in the management algorithm of early rectal cancer, at least as a diagnostic tool for a whole lesion biopsy in marginal cases. In this study, lesion size, positive lateral margins, high Bd, and type of knife used were associated with recurrence, thus implying a potential benefit even for patients with larger lesions. This observation, however, needs further evaluation in larger studies with longer follow-up, assessing more variables and long-term survival rates.

## Figures and Tables

**Table 1:** Patient demographics.

Variable	N = 51
Age (mean ± SD)	65.0 ± 11.1
Sex	
Female	21 (41.2%)
Male	30 (58.8%)
ASA score	
1	16 (31.4%)
2	23 (45.1%)
3	11 (21.6%)
4	1 (2.0%)
Reason for endoscopic resection	
Diagnosis/Staging	38 (74.5%)
Patient preference	9 (17.6%)
Unsuitable for surgery	4 (7.8%)
Antiplatelet or anticoagulation	6 (11.8%)

**Table 2:** Lesion characteristics.

Characteristic	N = 51 ^1^
Distance to the dentate line (cm)	3.9 ± 3.6
Part of the rectum	
Lower rectum	30 (58.8%)
Middle rectum	13 (25.5%)
Upper rectum	8 (15.7%)
Location	
Anterior	35 (68.6%)
Posterior	16 (31.4%)
Lesion Size (mm)	44.9 ± 25.9
Histology prior resection	
LGD adenoma	4 (7.8%)
HGD adenoma	19 (37.3%)
Intramucosal cancer	12 (23.5%)
Infiltrative cancer	4 (7.8%)
No biopsy	12 (23.5%)
Paris-Classification	
IIa	7 (13.7%)
IIa + c	10 (19.6%)
IIa + Is	22 (43.1%)
Is	12 (23.5%)
LST-Classification	
G-type Homogenous	8 (15.7%)
G-type Mixed	24 (47.1%)
NG-type Flat-elevated	2 (3.9%)
NG-type Pseudodepressed	7 (13.7%)
non LST	10 (19.6%)
JNET-Classification	
2A	6 (11.8%)
2B	35 (68.6%)
3	10 (19.6%)
Depression	16 (31.4%)
Ulceration	8 (15.7%)

^1^ Mean ± SD.

**Table 3:** Resection characteristics.

Characteristic	N = 51 ^1^
Duration (min)	185.8 ± 135.7
Method of resection	
ESD	39 (76.5%)
EID	8 (15.7%)
Partial EFTR	1 (2.0%)
Complete EFTR	3 (5.9%)
Plane of resection	
Submucosal	39 (76.5%)
Intermuscular	8 (15.7%)
Partial full-thickness	1 (2.0%)
Complete full-thickness	3 (5.9%)
Type of knife	
Needle-type knife (Dual knife, Flush knife, Hybrid knife)	39 (76.5%)
Needle-type + Hook knife	7 (13.7%)
Needle-type + IT knife	5 (9.8%)
Resected specimen size (mm)	61.1 ± 28.4
Macroscopic complete—En bloc resection	50 (98%)
Histological type	
Classical adenocarcinoma	47 (92.2%)
Mucinous adenocarcinoma	2 (3.9%)
Signet-ring carcinoma	2 (3.9%)
Histology-stage	
pT1bSM1	8 (15.7%)
pT1bSM2	20 (39.2%)
pT1bSM3	13 (25.5%)
Superficial pT2	8 (15.7%)
Deep pT2	2 (3.9%)
Lymphovascular invasion	16 (31.4%)
Perineural Invasion	3 (5.9%)
Budding score	
Bd1	29 (56.9%)
Bd2	11 (21.6%)
Bd3	11 (21.6%)
Differentiation	
G1	10 (19.6%)
G2	30 (58.8%)
G3	10 (19.6%)
G4	1 (2.0%)
Type of resection	
R0	31 (60.8%)
R1	19 (37.3%)
R2	1 (2.0%)
Lateral margins	
Clear	48 (94.1%)
Positive-adenoma	2 (3.9%)
Positive-carcinoma	1 (2.0%)
Vertical margins	
Clear	32 (62.7%)
Positive-adenoma	8 (15.7%)
Positive-carcinoma	11 (21.6%)
Depth of invasion from muscularis mucosa (μm)	2175.6 ± 932.3
Complications	9 (17.6%)

^1^ Mean ± SD.

**Table 4:** Follow-up.

Characteristic	N = 51 ^1^
Follow up (months)	20.6 ± 15.8
Endoscopic recurrence	7 (13.7%)
Type of endoscopic recurrence	
Adenoma	3 (5.9%)
Carcinoma	4 (7.8%)
No recurrence	44 (86.3%)
Treatment of endoscopic recurrence	
Endoscopic follow-up	1 (2.0%)
Endoscopic resection	5 (9.8%)
Surgery	1 (2.0%)
No recurrence	44 (86.3%)
Time of recurrence (months)	
3	3 (42.8%)
4	1 (14.3%)
6	1 (14.3%)
19	1 (14.3%)
24	1 (14.3%)
MRI-rectum protocol in follow-up	
Clear	23 (45.1%)
Not performed	23 (45.1%)
Recurrence	5 (9.8%)
Abdomen CT-scan in follow-up	
Clear	35 (68.6%)
Distant metastasis	2 (3.9%)
Lymph-node metastasis	1 (2.0%)
Not performed	13 (25.5%)
Adjuvant treatment options	
Chemotherapy	6 (11.8%)
Combined CRT	20 (39.2%)
Radiotherapy	25 (49.0%)
Adjuvant chemotherapy	26 (51.0%)
Type of chemotherapy	
5-FU	4 (7.8%)
5-FU with Leucovorin	5 (9.8%)
Capecitabine	15 (29.4%)
Capecitabine→FOLFIRI	1 (2.0%)
Capecitabine→FOLFIRI + Bevacizumab	1 (2.0%)
None	25 (49.0%)
Adjuvant Radiotherapy	45 (88.2%)
Type of radiotherapy (Gy dosage)	48 ± 1.5
Complication from adjuvant therapy	
No	47 (92%)
Radiation proctitis	2 (3.9%)
Bleeding	1 (2.0%)
Stricture	1 (2.0%)
Reason for no surgical treatment	
MDT proposal due to comorbidities	22 (43.1%)
Patient willingness	29 (56.9%)

^1^ Median (IQR).

**Table 5:** Significant variables in comparison to the presence of endoscopic recurrence.

	Endoscopic Recurrence		
Variable	No, N = 44 ^1^	Yes, N = 7 ^1^	*p*-Value ^2^
Reason for endoscopic resection			0.089
Diagnosis/Staging	33 (75%)	5 (71%)	
Patient preference	9 (20%)	0 (0%)	
Unsuitable for surgery	2 (4.5%)	2 (29%)	
Type of knife			**0.001**
Needle-type + Hook knife	7 (16%)	0 (0%)	
Needle-type + IT knife	1 (2.3%)	4 (57%)	
Needle-type knife (Dual knife, Flush knife, etc.)	36 (82%)	3 (43%)	
Paris-Classification			0.068
IIa	4 (9.1%)	3 (43%)	
IIa + c	10 (23%)	0 (0%)	
IIa + Is	20 (45%)	2 (29%)	
Is	10 (23%)	2 (29%)	
Preoperative MRI rectal-protocol staging			0.087
cT1	8 (18%)	0 (0%)	
Superficial cT2	8 (18%)	2 (29%)	
Deep cT2	2 (4.5%)	0 (0%)	
cT3a	1 (2.3%)	2 (29%)	
Not performed	25 (57%)	3 (43%)	
Budding score			**0.023**
Bd1	28 (64%)	1 (14%)	
Bd2	8 (18%)	3 (43%)	
Bd3	8 (18%)	3 (43%)	
Lateral margins			**0.046**
Clear	43 (98%)	5 (71%)	
Positive-adenoma	1 (2.3%)	1 (14%)	
Positive-carcinoma	0 (0%)	1 (14%)	
MRI-rectum protocol in follow-up			**<0.001**
Clear	22 (50%)	1 (14%)	
Not performed	22 (50%)	1 (14%)	
Recurrence	0 (0%)	5 (71%)	
Abdomen CT-scan in follow-up			**0.025**
Clear	30 (68%)	5 (71%)	
Distant metastasis	1 (2.3%)	1 (14%)	
Lymph-node metastasis	0 (0%)	1 (14%)	
Not performed	13 (30%)	0 (0%)	
Adjuvant chemotherapy	20 (45%)	6 (86%)	0.10
Adjuvant Radiotherapy	39 (89%)	6 (86%)	>0.9
Adjuvant treatment options			0.10
Chemotherapy	5 (11%)	1 (14%)	
CRT	15 (34%)	5 (71%)	
Radiotherapy	24 (55%)	1 (14%)	
Lesion Size (mm)			**0.011**
40 or more	20 (45%)	7 (100%)	
Less than 40	24 (55%)	0 (0%)	

^1^ *n* (%); ^2^ Fisher’s exact test.

**Table 6:** Binary regression analysis.

Variable	OR	*p*-Value
Clear vertical margins		1
Positive margins for adenoma	0.003	1
Positive margins for carcinoma	0.000	1
Depth of invasion more than 2000 μm	0.718	0.83
R0 resection	0.001	1
Budding (Bd1)		0.56
Budding (Bd2)	9.351	0.22
Budding (Bd3)	19.081	0.16
Differentiation (G1)		0.88
Differentiation (G2)	0.260	0.58
Differentiation (G3)	0.138	0.42
Differentiation (G4)	0.000	1
Lymphovascular invasion	0.359	0.53
pT1bSM1		0.99
pT1bSM2	0.756	0.89
pT1bSM3	1.163	0.95
Superficial pT2	0.000	0.99

## Data Availability

The raw data generated from this study are available upon request to the corresponding author.
